# Spatial Proteomics Reveals Alzheimer’s Disease–Specific Human Microglial States

**DOI:** 10.1002/alz70861_109002

**Published:** 2025-12-23

**Authors:** Bryan Cannon

**Affiliations:** ^1^ Stanford University, Palo Alto, CA USA

## Abstract

**Background:**

Microglia contribute to aging, neurodegeneration, and Alzheimer’s disease (AD), but their diversity and interactions within the human brain remain poorly characterized due to limitations of low‐plex protein imaging and differences from rodent models.

**Method:**

Using Multiplexed Ion Beam Imaging (MIBI), we spatially mapped cellular states and microenvironments in cognitively normal (CN) human brains, identifying a spectrum of proteomic microglial profiles across ten individuals. Single‐nucleus assay for transposase‐accessible chromatin sequencing (snATAC‐seq) was used in parallel to validate molecular functions associated with these microglial states.

**Result:**

Microglial immune activation states varied across brain regions and microenvironments, revealing proteomic trends among CN individuals. In AD tissues, microglia exhibited significant shifts at the immunologically active end of the proteomic spectrum, including increased expression of CD33 and CD44 and decreased expression of HLA‐DR, P2RY12, and ApoE.

**Conclusion:**

These findings establish an in situ, single‐cell spatial proteomic framework that defines Alzheimer’s disease‐specific microglial states and regulatory alterations.

